# Recent Progress in Birdcage RF Coil Technology for MRI System

**DOI:** 10.3390/diagnostics10121017

**Published:** 2020-11-27

**Authors:** Sheikh Faisal Ahmad, Young Cheol Kim, Ick Chang Choi, Hyun Deok Kim

**Affiliations:** 1 Institute of Advanced Convergence Technology, Kyungpook National University, 80 Daehak-ro, Buk-gu, Daegu 41566, Korea; faisalthestar@knu.ac.kr (S.F.A.); yckim@knu.ac.kr (Y.C.K.); choic@knu.ac.kr (I.C.C.); 2 School of Electronics Engineering, College of IT Engineering, Kyungpook National University, 80 Daehak-ro, Buk-gu, Daegu 41566, Korea

**Keywords:** birdcage coil, RF coil, volume coil, numerical method, analytical solution, electromagnetic simulation, MRI, NMR imaging, design issues, dual resonance

## Abstract

The radio frequency (RF) coil is one of the key components of the magnetic resonance imaging (MRI) system. It has a significant impact on the performance of the nuclear magnetic resonance (NMR) detection. Among numerous practical designs of RF coils for NMR imaging, the birdcage RF coil is the most popular choice from low field to ultra-high field MRI systems. In the transmission mode, it can establish a strong and homogeneous transverse magnetic field B1 for any element at its Larmor frequency. Similarly, in the reception mode, it exhibits extremely high sensitivity for the detection of even faint NMR signals from the volume of interest. Despite the sophisticated 3D structure of the birdcage coil, the developments in the design, analysis, and implementation technologies during the past decade have rendered the development of the birdcage coils quite reasonable. This article provides a detailed review of the recent progress in the birdcage RF coil technology for the MRI system.

## 1. Introduction

Nuclear magnetic resonance (NMR) imaging is considered to be the most advanced and comprehensive analysis technique in various research fields of modern science. The NMR phenomenon was firstly introduced in 1936 in order to measure the nuclear magnetic moments of various nuclei and neutrons [1,2,3,4]. The method is based on the detection of the electromagnetic waves with specific frequencies that are emitted from the pre-exited hydrogen (^1^H) protons in a strong and homogeneous magnetic field. For a long period of time, the main use of NMR was limited to spectroscopic analysis in chemistry and physics. However, NMR spectroscopy was not restricted to the ^1^H proton; it was extended to element with unpaired proton, such as ^13^C, ^14^N, ^17^O, ^19^F, ^23^Na, and ^31^P [5]. In the early 1970′s, the use of NMR for clinical medical imaging applications was proposed [6,7,8]. However, the methods for using the NMR technique for medical imaging with some appropriate instrumentation, which is currently known as the magnetic resonance imaging (MRI) system, for clinical use were demonstrated in the late 1970′s and early 1980′s [9,10,11,12]. MRI is a nonradiative and noninvasive procedure that is performed to obtain detailed information on the internal metabolism of the biological objects, such as human and animal, in the form of two-dimensional (2D) grayscale images. This feature has made MRI a preferable choice for medical imaging for the last three decades, even in the presence of some excellent medical imaging techniques, such as X-ray, ultrasonography, computed tomography (CT), positron emission tomography (PET), etc.

Among various parts of the MRI system, radio frequency (RF) coils have gained more attention, owing to their direct involvement in the RF signal transmission and reception [13]. RF coils establish a purely homogeneous alternating magnetic field (known as the B1 field) in the direction transverse to the main static magnetic field (known as the B0 field) of the MRI system. The B1 field excites the unpaired protons at a specific radio frequency that is known as the Larmor frequency [4,9]. This process increases the energy level of the unpaired protons, which enables them to move to an unstable higher energy state. Upon the removal of the B1 magnetic field, the protons release the absorbed energy at the same Larmor frequency and return to their original stable energy state. The released energy is known as the Field Induction Delay (FID) which will refer as the NMR signal in the manuscript. Such a signal is received or detected by the same transmitting RF coil or a separate receiver RF coil. RF coil can be designed for the detection of NMR signal either from the whole volume or from a certain location of the object. The NMR signal is a faint signal that emerges at a short time interval [9]. This faint NMR signal can be detected by a well-designed, highly sensitive RF coil with a higher signal-to-noise ratio (SNR), and it is further converted to the meaningful images while using signal and image processing techniques. However, an RF coil that is designed at a specific NMR frequency of a certain element cannot be used for any other element or MRI system with different field strength.

There have been multiple designs of the RF coils for various applications [14,15]. Moreover, there are multiple methods for categorizing these RF coils on the basis of their certain characteristic. When considering their operating principles, the RF coils can be categorized into the transmitter RF coil, receiver RF coil, and transceiver RF coil [10,13]. The transmitter RF coil is only used for the establishment of a homogeneous B1 magnetic field in the imaging volume at Larmor frequency whereas the receiver coil with high SNR is used only for the detection of the NMR signal at the same Larmor frequency. Conversely, the transceiver RF coil is used to perform both transmission and reception operations. Based on the structural geometry of RF coils, they can be divided into the volume coils and surface coils [16]. Generally, the volume coils are mostly designed in the cylindrical shapes and they are used to obtain the NMR images of the whole subject. The saddle coil [17], birdcage coil [18], transverse electromagnetic (TEM) coil [19], and phased array coil [20] are some famous and widely used volume coils. On the other hand, the surface coils are considered to be the 2D planner version of RF coils [21,22]. They are used to obtain the NMR images of a specific location of interest from the whole volume. In the viewpoint of the polarization of alternating magnetic field B1, RF coils are categorized into linearly and circularly polarized types [13]. The linearly polarized RF coils are equipped with a single port, whereas the circularly polarized RF coils are equipped with two ports (in phase quadrature to each other) for the transmission and/or reception of the RF signal. An RF coil with a circular polarization has √2 times higher SNR than its linearly polarized version [23]. When considering the direction of the magnetic field B1, RF coils can be classified into the axial field RF coil and the transverse field RF coil [24]. The axial field RF coils are used in order to produce the B1 magnetic field in the direction parallel to their axis of symmetry, whereas the transverse field RF coils are used to produce the B1 magnetic field in the direction perpendicular to their axis of symmetry.

The RF coils used for MRI are, in fact, microwave resonators that are capable of producing a homogeneously distributed strong near magnetic field (B1) [25]. These are composed of inductive and capacitive elements with ideally zero resistance for producing the desired resonance frequency [15]. Conventionally, the design and analysis process of RF coils is performed while using the microwave circuit analysis techniques that are simple to use. However, their accuracy is limited by two factors. First is the lack of precision in the establishment of the electrical equivalent circuit model of the radiating structure. The second, which is more important, is their incompetency to properly discretize the region around the radiating structure for the analysis of electromagnetic (EM) fields. The other obvious solution for the design and analysis of RF coils is the complex analytical numerical methods, such as finite element method (FEM), finite difference time domain (FDTD) method, and method of moments (MoM). These methods provide a more accurate design and analysis of RF coil, as they address the above described second factor quite efficiently. However, they involve a complex numerical computation that requires extensive mathematical knowledge. In the previous decade, the advancement and progress in the 3D full-wave EM simulation software have completely changed the trends in the field of RF coil design. These software, which are based on the analytical numerical methods, provide a single platform for the design and analysis process of the RF coils.

The implementation of RF coils is a sensitive and crucial procedure. The type and geometry of the conductor, the self and mutual inductances, and the lumped capacitance are the factors that need to be seriously considered. The introduction of flexible printed circuit board (FPCB) etched conductors to the implementation process of RF coil have revolutionized the field of RF coil engineering [26]. RF coils implemented while using such techniques are more mechanically robust, less immune to circuit losses, and they exhibit excellent performance.

The birdcage RF resonator is the most famous and widely used RF coil for the volume NMR imaging for clinical and preclinical applications [27]. The TEM and phased array coils are also the prominent candidates for volume NMR imaging applications. However, they require rigorous design with respect to the target applications, as well as the complex electronic circuitry to control the transmitted and received RF signal, which somewhat turn into a disadvantage. On the other hand, the birdcage coil is known for producing the strong and homogeneous magnetic field, regardless of the coil size, target application, and field strength of the MRI system. In this paper, a comprehensive review of the progress in the design, analysis, and implementation methods of the birdcage coil is presented. Aside from reviewing the development of the conventional techniques, the main motive of this work is to focus on the recent trends that have been adopted in the previous decade for the design, analysis, and implementation of the birdcage coil for various MRI applications. This paper is divided into three parts. The first part is about the “Birdcage Coil”, which is dedicated to the literature regarding the principle and functionality of the conventional birdcage coil. The second part presents a comprehensive review of the work reported in the field of “Design and Analysis of the Birdcage Coil”. The last part presents the literature review of the “Implementation Techniques of the Birdcage Coil”.

## 2. Birdcage Coil

The birdcage coil (or resonator) was first proposed in 1985 by Hayes et al. [18]. The name birdcage was coined, owing to the structural resemblance of the birdcage coil to the traditional hanging-type cage for birds. The birdcage coil is a transverse field resonator that was proposed to resolve the issues that were observed in the famous saddle coil [17]. The birdcage coil is considered to be the most successful design for NMR imaging applications, owing to its stronger B1 magnetic field homogeneity and higher SNR. It is a volume-type RF resonator with a cylindrical shape, and it can be designed in any size for clinical and nonclinical applications at low field, high field, and ultra-high field MRI systems. The conventional birdcage coil is composed of an even number of straight conductor segments of similar dimensions that are known as legs (or rungs). In order to realize a cylindrical structure, these legs are short-circuited on both sides with similar conductor rings known as end-rings.

### 2.1. Types of the Birdcage Coil

**Figure 1:**
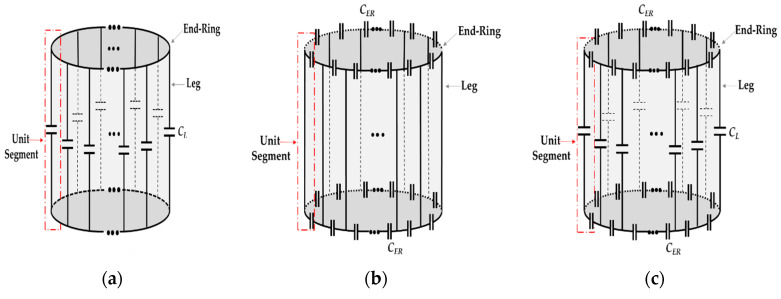
Schematic diagrams of the various types of the birdcage coil; (**a**) Low pass; (**b**) High pass; and, (**c**) Band pass.

The birdcage coil is an LC resonator in which the inductance is provided by the conductor segments, whereas the lumped capacitors (with nonmagnetic characteristics) are used to fulfill the needs of the capacitance. Based on the location of these lumped capacitors in electrical circuit of the coil, three types (or configurations) of the conventional birdcage coil can be defined, namely low pass (LP), high pass (HP), and band pass (BP), which are presented in Figure 1. In the LP birdcage coil, the identical lumped capacitors *C_L_* are inserted into the legs, whereas the end-rings at both sides are completely short-circuited, as presented in Figure 1a. The birdcage coil supports multiple resonance frequency modes. The lowest leg current-based resonance frequency mode of the LP birdcage coil is used in NMR imaging applications. The HP birdcage coil is implemented by inserting the identical capacitors *C_ER_* into the end-ring segments between the conductive legs, as presented in Figure 1b. The highest leg current-based resonance frequency mode of the HP birdcage coil is used for NMR imaging applications. The BP birdcage coil is also known as the hybrid birdcage coil, as it contains the identical capacitors *C_L_* into the legs and *C_ER_* into the end-rings, as presented in Figure 1c. The BP birdcage coil can be designed in LP or HP mode according to the target application. In the LP mode, the lowest resonance frequency that is based on the leg current is used for NMR imaging, whereas the highest one is used in the HP mode. The BP birdcage coil is known to provide better control of the resonance frequency modes when compared with the other two types. However, it renders the appropriate designing of BP birdcage little more crucial because of tightly spaced resonance frequency modes [28].

### 2.2. Resonance Modes of the Birdcage Coil

#### 2.2.1. Resonance Frequencies

In the first article regarding the birdcage coil, Hayes et al. described that a birdcage coil with *N* number of legs and two end-rings can support maximum *N*/2 +1 resonance frequency modes [18]. The occurrence of *N*/2 resonances is due to the formation of unique *N*/2 closed current loops involving the legs and the end-ring segments, whereas the occurrence of the only remaining resonance is caused by the end-rings current. At a specific frequency among the *N*/2 resonance frequency modes, the magnitude of current on each leg of the birdcage coil varies in a sinusoidal (or cosine) manner. This resonance frequency mode is known as the dominant resonance mode (or the lowest-impedance mode). It is responsible for the homogeneous distribution of B1 magnetic field in the transverse plane of the birdcage coil. The other leg current-based resonance frequency modes are known as the higher-order modes (or higher-impedance modes). Generally, the higher-order resonance modes are not used for NMR imaging, due to their non-homogeneous magnetic field profile inside the coil. However, few birdcage coils have been reported where the researchers used these higher-order modes for parallel imaging or dual-resonance NMR applications [29,30,31,32,33]. Jin et al. conducted a linear circuit analysis (LCA) and presented a compact analytical expression, as provided in Equation (1), which is used in order to calculate all of the *N*/2 +1 resonance frequency modes of the birdcage coil [34].

(1) $$\begin{array}{c} \omega_{m}=\sqrt{\frac{\frac{2}{C_{L}}\sin^{2}\frac{m\pi }{N}+\frac{1}{C_{ER}}}{L_{L}\sin^{2}\frac{m\pi }{N}+L_{ER}}}\\ \left(m=0,1,2,\ldots ,N/2\right) \end{array}$$

Here, *L_L_* and *L_ER_* denote the equivalent self-inductances of the leg and end-ring segment, whereas *C_L_* and *C_ER_* denote the lumped capacitors used in the legs and end-ring segments of the birdcage coil, respectively. In [34], a more detailed procedure, which also involves the effect off mutual inductances of the legs and end-ring segments, can be found. Equation (1) presents a generalized expression for the evaluation of the resonance frequency modes of BP birdcage coil. It can be modified for the LP and HP birdcage coils by removing the terms that contain the leg capacitor *C_L_* and the end-ring capacitor *C_ER_*, respectively. In Equation (1), the resonance mode *ω*_0_ is the resonance of the end-rings of the birdcage coil. Initially, it was assumed that only one resonance frequency mode is possible due to the current flowing through the end-rings of birdcage coil. However, with the help of the circuit theory and discrete Fourier transform (DFT), Leifer et al. revealed that a birdcage coil with *N* number of legs can support maximum *N*/2 + 2 resonance frequencies [35]. Moreover, he demonstrated that, along with the traditional anti-rotating end-ring resonance frequency mode *ω_AR_* in the birdcage coil, a co-rotating end-ring resonance frequency mode *ω_CR_* can also simultaneously exist. These resonances can be determined while using the following equations:

(2) $$\omega_{AR}=\sqrt{\frac{N}{C_{ER}\left(L_{ER}-M_{ER}\right)}}$$

(3) $$\omega_{CR}=\sqrt{\frac{N}{C_{ER}\left(L_{ER}+M_{ER}\right)}}$$

The term *M_ER_* used in Equations (2) and (3) denote the mutual inductance of the end-ring segments. The end-ring resonance only occurs in the HP and BP configurations of the birdcage coil, not in the LP configuration due to of the absence of the lumped capacitors in the end-rings. Because the end-ring current produces a magnetic field parallel to axis of symmetry of the birdcage coil, it is therefore not favorable to use the end-ring resonance mode for NMR imaging.

#### 2.2.2. Dominant Resonance Path

**Figure 2:**
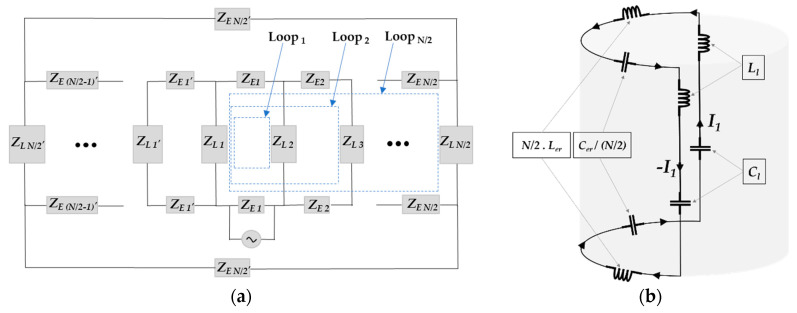
Schematic diagrams of the equivalent circuit of the birdcage coil showing. (**a**) the closed loops for leg currents; (**b**) dominant Resonance Path [36].

The conventional birdcage coil is composed of several closed current loops, as presented in Figure 2a. Each loop has its own specific resonance frequency that is regarded as the resonance frequency mode of birdcage coil. In a recent work, Kim et al. employed an intuitive approach and introduced the concept of dominant resonance path in order to determine the closed current loop in the birdcage coil, which is responsible for establishing the dominant resonance frequency mode [36]. The dominant resonance path, as presented in Figure 2b, is the longest closed loop (Loop _N/2_) in the birdcage coil that contains two legs that are connected to each other through the *N*/2 consecutive segments of both end-rings. This concept is useful for determining the lumped capacitance of the birdcage coil.

## 3. Design and Analysis of the Birdcage Coil

The birdcage coil is widely known for its homogeneous magnetic field characteristics. Thus, it is necessary to have an appropriate design and careful analysis of the birdcage coil, as B1 magnetic field that is extremely high causes bright spots and B1 magnetic field that is extremely low causes dark spots in the obtained NMR images [37]. For the design and analysis of the birdcage coil, analytical solutions are usually obtained by using various theoretical techniques. The main outcomes of this process are the resonance frequency modes, B1 magnetic field distribution in the imaging sample volume, specific absorption rate (SAR) of the biological object, port impedance characteristics for external circuit interfacing, and return loss (S11) at the dominant resonance frequency. The theoretical methods that are employed for the design and analysis of the conventional birdcage coil are either analytical or numerical. The following section presents a brief review of the literature on the use of the theoretical methods for birdcage coil.

### 3.1. Analytical Methods

The analytical methods are based on the solution of circuit equations which are defined by using the electrical equivalent circuit model. Because of the microwave structure of the birdcage coil, it can be modeled while using lumped elements or transmission lines. A generalized analytical solution can be devised for both cases by using either the linear circuit analysis (LCA) or transmission line analysis (TLA) accordingly. These analytical solutions are mostly adopted in order to predict the resonance frequency spectrum and impedance characteristics of the birdcage coil. Some analytical solutions can be extended to determine the currents on the coil legs that are used to compute the distribution of B1 magnetic field.

#### 3.1.1. Linear Circuit Analysis (LCA)

Based on the LCA, Hayes et al. devised the very first analytical solution for the prediction of the resonance frequency modes of the LP birdcage coil [18]. They used Biot–Savart law to explain the of B1 magnetic field distribution; however, they did not provide any mathematical expression for this. Tropp et al. also employed the LCA and developed an analytical solution for the resonance frequency modes of the LP birdcage coil [38]. He solved the Kirchhoff voltage law-based mesh equations in the frequency domain using the Laplace transform. Moreover, he conducted an intensive theoretical investigation in order to explain the perturbations in the birdcage coil due to inductive or capacitive coupling. In another LCA based analytical method, Joseph et al. constructed a model for the analysis of the HP birdcage coil [39]. They established an analytical solution for the evaluation of mutual inductances in the birdcage coil and included it in the Kirchhoff voltage law-based mesh equation. He used the DFT of the currents with respect to the angular position of the legs and predicted the resonance frequency modes of the HP birdcage coil. Leifer et al. proposed a more comprehensive and generalized analytical solution by employing the LCA [35]. This analytical solution could be used in order to predict the resonance frequency modes for all types of the birdcage coil. He solved the Kirchhoff voltage law-based mesh equations, which also include the mutual inductance in the frequency domain by using DFT. His proposed analytical solution could also be used in reverse to provide the inductances of the coil by applying the inverse DFT to the mesh equations in combination with the measured resonance frequency modes. In another useful work, Giovannetti et al. adopted the LCA in combination with the magnetostatic theory and developed a software for evaluating the resonance frequency modes and plotting the B1 magnetic field distribution of the LP and HP birdcage coils [40]. Moreover, Novikov et al. used LCA and conducted a detailed analysis of the BP birdcage coil. He also included the physical resistance of the conductors, along with mutual inductance in the Kirchhoff voltage law-based mesh equation [41]. Contrary to the abovementioned previous works, he conducted the time domain analysis and adopted Green’s function theory in order to propose a more generalized analytical solution. His analysis technique could also be used to determine the active resistance, which is a useful parameter for the evaluation of various power losses that are related to the birdcage coil. Most of the LCA-based analytical methods described in this section can be used to calculate the currents on the birdcage coil legs that are further used to determine the magnetic field distribution of the birdcage coil. Benyahia et al. provided the Biot–Savart law-based analytical equations to compute the B_1_ magnetic field components of a birdcage coil [42]. They used the same technique to determine the leg currents that was described by Boissoles et al. [43].

#### 3.1.2. Transmission Line Analysis (TLA)

The TLA method was first adopted by Watkins et al. for the design and analysis of the HP birdcage coil [44]. They produced a similar analytical solution for resonance frequency modes that was presented earlier by Hayes et al. while using the LCA. Pascone et al. proposed a more detailed solution for the LP and HP configurations of the birdcage coil using the TLA [45]. They used the transmission matrix (ABCD matrix) theory and proposed an analytical solution that relates the input impedance of the birdcage coil with its lumped parameters to efficiently predict the resonance frequency modes. Their proposed method also theoretically elucidated the interfacing of any external circuit at the end-ring of the birdcage coil. Kim. et al. provided a more comprehensive analytical solution for the BP configuration of the birdcage coil while using the TLA [46]. He also used the transmission matrix theory and proposed a more generalized analytical solution that can be easily modified for the conventional LP and HP and even more complex configurations of the birdcage coil. His proposed analysis method also theoretically explained the interfacing of external circuits in end-rings and legs. In another work, Abbas et al. adopted the TLA and developed an analytical solution in order to determine the B_1_^+^ field distribution for the TEM coils [37]. Subsequently, he modeled the birdcage coil as a transmission line and successfully developed an analytical solution for its B_1_^+^ field distribution using the TLA.

#### 3.1.3. Limitations of the Analytical Methods

The analytical methods are widely used in the design and analysis of the birdcage coil. However, the accuracy of these methods is questioned due to several limitations. Some of these limitations, such as the mutual inductance, physical resistance of the coil conductive circuits, skin effect at microwave frequencies, and the physical orientation of the lumped elements, are related to the physical structure of the birdcage coil. There are some other factors, such as SNR, SAR, and B1 magnetic field distribution, whose exact knowledge in the presence of heterogeneous biological sample cannot be accurately predicted while using the analytical methods.

### 3.2. Numerical Methods

The numerical or computational methods are the best alternative to the analytical methods. The use of numerical analysis techniques, such as FEM, FDTD, and MoM, for the analysis and design of the radiating structures has been a common practice in electromagnetics. These numerical methods are used for discretizing the radiating structures and computing the EM fields by using the well-known Maxwell equations in combination with the computational EM field theory. These field quantities are further used for determining the other parameters that are related to the analysis and design process. Similar to other microwave structures, these numerical methods are equally beneficial for the precise and accurate design and analysis of the birdcage coils. They are used in two different ways, as explained in the following subsections.

#### 3.2.1. Theoretical Modeling Techniques

The theoretical modeling techniques are based on the computational techniques that require excellent mathematical knowledge and good computer programing skills. These techniques are employed in the birdcage coil for the computation of the EM fields. The B1 magnetic field distribution is the main parameter of interest in the birdcage coil, whereas the electric field is important, as it is used to calculate the SAR. These techniques can also be used to predict the resonance frequency modes. Jin et al. developed a hybrid theoretical modeling technique and combined it with the biconjugate gradient algorithm of MoM with fast Fourier transform to compute the EM fields and SAR of various linear and quadrature birdcage coils [47]. Jin et al. also employed the FEM based theoretical modeling technique for the analysis of the birdcage coil that was loaded with a human head phantom [48]. They calculated the B1 magnetic field and SAR distributions at 64, 128, 171, and 256 MHz for the linear and quadrature configurations of the BP birdcage coil. Chen et al. developed a hybrid computational technique by combining the MoM and the FDTD method for the analysis of a shielded birdcage coil for the ^1^H proton MRI at various frequencies [49]. They employed MoM in order to determine the currents on the legs of the birdcage coil and then the FDTD method to compute the related EM fields while using those currents. Jiao et al. proposed a fast frequency-sweep technique using the MoM. A very fast analysis for determining the resonance frequency modes of the birdcage coil could be performed using this technique [50]. Ibrahim et al. proposed an algorithm using the FDTD method for the theoretical modeling of the birdcage coil [51]. They included the coupling between the resonator and sample in order to develop more realistic analytical solutions for the currents that can precisely evaluate the EM fields.

#### 3.2.2. 3D Electromagnetic Simulations

However, although the theoretical modeling methods provide accurate and precise analysis of the birdcage coil, they involve very tedious mathematical work. The introduction of the commercially available full-wave EM simulation software has made the design and analysis process of the birdcage coil much easier. These simulation software, which are based on the theoretical computational methods, such as FEM, FDTD, and MoM, provide a convenient user interface, thus eliminating the use of additional computer programming [26]. These software also provide important features, such as the cad tool, to draw the exact 3D model of the birdcage coil, the built-in libraries with a wide range of materials for dielectric substrates and conductors, the lumped elements defining capabilities, and many different excitation sources with editable boundary conditions with multiple other features at a single platform. The most important feature of these software tools is the post processing option that provides the final desired results, such as return loss, B1 magnetic field distribution, and SAR. Almost all software includes the phantoms of human and animal in their material libraries, which facilitates the design of the birdcage coils with maximum precision and accuracy. Nowadays, most commercially available EM simulation software also provide special dedicated libraries for the design and analysis of birdcage coils. Most of the work regarding the birdcage coil reported in the past decade is based on the full-wave 3D EM simulations, owing to the availability of such software. Table 1 presents a detailed review of the literature regarding this work.

#### 3.2.3. Comparison of the Simulation Techniques

Based on Table 1, it can be concluded that the FEM-based software (HFSS and COMSOL) and FDTD-based software (XFDTD and CST) are widely in use for the designing and analysis process of the birdcage coil. The FEM-based full-wave simulation software are preferred, owing to their capability to efficiently mesh the complex geometries. Their only disadvantage is the requirement of large computational resources to solve large-sized equations matrix in the post processing. Conversely, the FDTD-based software are very simple and efficient to use. However, their method of meshing the complex geometries is not as good as that of the FEM-based software. This can cause significant errors in the design and analysis process. Li et al. provided a detailed review of the numerical techniques that were used for the analysis of RF coils [85]. They revealed that a hybrid numerical technique, i.e., simultaneous use of two numerical methods, can provide more accurate analysis of RF coils. Nowadays, most of the modern numerical simulation software employs the hybrid numerical simulation techniques in order to achieve a fast, efficient, and accurate analysis.

## 4. Implementation Techniques of the Birdcage Coil

The modern 3D full-wave EM simulation software have made it possible to design the birdcage RF coil with various sizes for different clinical and nonclinical applications. The designs of the birdcage coil that were obtained via numerical simulations under appropriate initial conditions and simulation setup are quite ideal, as there are several physical parameters that cannot be properly modeled. The desired performance characteristics of the birdcage coil can only be achieved if the prototype of the birdcage coil is perfectly implemented [27]. Because the MRI is performed at microwave radio frequencies, the geometry of the conductor, the self and mutual inductances, and the lumped capacitors are the critical parameters in proper implementation of the birdcage coil. These factors not only affect the SNR, but also the homogeneous distribution of the B1 magnetic field at the dominant resonance frequency. A review of the literature, which highlighted the different techniques for the implementation of the birdcage coil, is provided in the following subsection.

### 4.1. Basic Implementation Considerations

#### 4.1.1. Conductor Geometry

**Figure 3:**
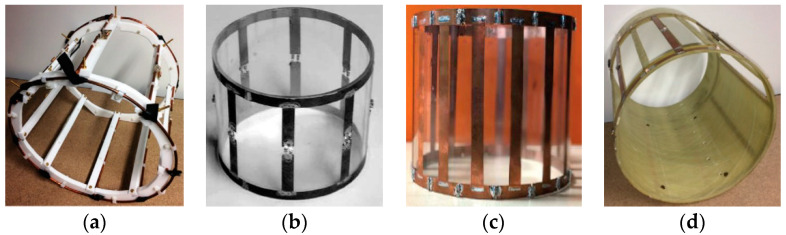
Birdcage coil implemented with different conductors: (**a**) Cylindrical wire [86]; (**b**) Rectangular strip [40]; (**c**) Rectangular foil [77]; and, (**d**) Rectangular foil etched on a flexible printed circuit board [86].

The electrical circuit of the birdcage coil is composed of ideally lossless conductors and nonmagnetic capacitors. The conductors that are used as the legs and/or end-rings of the birdcage coil are usually cylindrical wires, rectangular strips, and rectangular foils (micrometer thickness), as presented in Figure 3.

Ideally, the direct current (dc) resistance of the conductors in the birdcage coil is considered to be zero. However, the skin effect phenomenon at microwave frequencies becomes a source of alternating current (ac) resistance in these conductors. The choice of conductor depends on the field strength of the MRI system and its corresponding resonance frequency. For the lower field (<0.5T) birdcage coils, Giovannetti et al. presented a detailed comparison between the rectangular and cylindrical conductors [87]. Through bench-testing, they proved that a birdcage coil built using the cylindrical conductor exhibits better performance than the one that was built using rectangular or foil conductors. They established a relationship between rectangular and cylindrical conductors. Such a relationship can be useful for building the birdcage with either cylindrical or rectangular conductors with nearly similar performance characteristics. For high field birdcage coils, the authors of this article have presented in a work published elsewhere that a coil that is made of a cylindrical conductor exhibits higher resistance at high field (>3T) imaging frequencies [88]. They also revealed that the RF coil that is made of foil conductors etched on a flexible dielectric substrate (or FPCB) exhibits excellent characteristics on high field MRI applications. For ultra-high field (>9.4T) birdcage coils, Xu et al. conducted an extensive study regarding the conductors with various shapes being used for the coil construction [58]. They used 12 conductors with different cross-sectional shapes and confirmed that the width of the leg conductors is an important factor in the performance of the birdcage coil.

#### 4.1.2. Inductance of the Birdcage Coil

The birdcage coil is an LC resonator. The conductors (either cylindrical wires or rectangular strips (or foils)), which are used as the legs and end-rings, are the source of distributed inductance in the birdcage coil. The self-inductance of the cylindrical conductor with length *l* and radius *r* and rectangular conductor with length *l* and width *w* can be determined while using the Equations (4) and (5), respectively [34].

(4) $$L=\frac{\mu_{o}l}{2\pi }\cdot \left[\ln\frac{2l}{r}-1\right]$$

(5) $$L=\frac{\mu_{o}l}{2\pi }\cdot \left[\ln\frac{2l}{w}+\frac{1}{2}\right]$$

The effective inductance of the birdcage coil is a combination of the self-inductance and mutual inductance of the conductor. The mutual inductance between two parallel conductors of length *l* and separated by a distance *d* are given by following equation [34]:

(6) $$M=\frac{\mu_{o}l}{2\pi }\cdot \left[\ln\left(\frac{l}{d}+\sqrt{1+\frac{l^{2}}{d^{2}}}\right)-\sqrt{1+\frac{d^{2}}{l^{2}}}+\frac{d}{l}\right]$$

However, the calculations of the mutual inductance are quite complex for the birdcage coil, as it is composed of *N* number of parallel legs and parallel end-ring segments. Some researchers developed the theoretical methods in order to determine the mutual inductance for the legs and the end-ring segments of the birdcage coil. In most of the solution, the mutual inductances of a conductor (leg or end-ring segment) are computed as eigenvalues of a circulant matrix that were obtained by solving the circuit analysis-based mesh equations in the time or frequency domains [35,39,43,89,90]. However, Pascone et al. proposed a solution for the mutual inductance that is based on the calculation of the distances between the parallel leg conductors while using the filamentary approximation and between the end-ring segments using the polygonal (octagonal) approximation [91].

#### 4.1.3. Capacitance of the Birdcage Coil

The capacitors used in the birdcage coil are lumped components. A nonmagnetic capacitor with a high Q-factor has been proven to increase the performance of the birdcage coil [36,87]. Usually, the approximate value of the capacitance is computed while using Equation (1) [18]. Kim et al. proposed a method for the determination of the capacitance for the leg and/or end-ring using the dominant resonance path concept [36]. In addition, Chin et al. employed the simple circuit theory technique of voltage drop across the leg and end-ring segments and determined the required capacitances for the legs and/or end-rings [90]. They used this technique in their famous software, named “Birdcage Builder”, which is commonly used for determining the leg and end-ring capacitances of the birdcage coils. The lumped components are known to execute the nonlinear characteristics at higher microwave frequencies, which can be a limiting factor for the use of birdcage coil in high field and ultra-high field applications. It has been demonstrated, by Hayes et al. for large-sized birdcage coils [27] and Son et al. for the small-sized birdcage coils [92], that the replacement of the single-leg capacitor with multiple series connected identical capacitors can improve the B1 magnetic field homogeneity along the axis of symmetry of the birdcage coil. Some birdcage coils, which were implemented without lumped capacitors, have also been reported. The capacitance that was required for the desired resonance in such birdcage coils was obtained virtually by overlapping a specified part of the conductor segments in the leg, end-ring, or leg and end-rings [74,93,94,95]. The capacitors that are implemented by such techniques are usually referred to as integrated capacitors.

### 4.2. Special Types of Birdcage Coil

Along with the conventional birdcage coil, which is designed in LP, HP, and BP configurations, the special types of the birdcage coil have also been presented. These types were either proposed for special MR imaging application or for the detection of the NMR signals from various elements while using the single birdcage coil. The modern EM simulation software have been successfully used in the realization of the complex designs of the birdcage coil, as previously mentioned. A brief literature review of some of the special designs of the birdcage coil is discussed in the following subsections.

#### 4.2.1. Modified Birdcage Coils

The birdcage coil with a different physical appearance in terms of the shape and leg conductor pattern or has any additional conductive section, which is part of the basic resonator that is referred to as the modified birdcage coil in this article. The main purpose of the modification is to improve the B1 magnetic field homogeneity and strength under general or special circumstances. Table 2 presents some featured modified designs of the birdcage coil.

#### 4.2.2. Dual-Resonant Birdcage Coils

The birdcage coil was originally designed for the single resonance operation with a unique dominant resonance frequency mode that is used for the MRI of the ^1^H proton [18]. Afterward, the birdcage coils were also developed for the MRI of element, such as, ^23^Na, ^31^P, and ^3^He, in the biological objects for clinical and nonclinical purposes [76,103,108,109]. Instead of using separate birdcage coils for the MRI of multiple elements, the dual-resonant birdcage coils were designed, which can perform the MRI of the ^1^H and another element without replacing the coil. Special techniques were also proposed in order to create two distinct leg current-based dominant resonance modes at the desired frequencies in a single birdcage coil. The dominant resonance frequency for the ^1^H proton MRI is always higher than the second dominant resonance frequency for the MRI of another element, as the gyromagnetic ratio γ of ^1^H proton is the highest (42.58 MHz/T) among those of the other elements that are found in biological objects. Table 3, below, presents some commonly used and newly proposed techniques for the dual-resonant operation using birdcage coil.

#### 4.2.3. Ultra-High Field Birdcage Coils

It is generally acknowledged that the quality of NMR images improves with the strength of the magnetic field. The Birdcage coil is also a potential candidate for ultra-high field (7T and above) MRI and it is used for the similar applications, as it is used for the high field (less than 7T) MRI. However, the design of the birdcage coils for ultra-high field MRI applications is a challenging task. There are several potential issues and challenging factors which can limit the use of the birdcage coil in high field MRI [116]. With the increase of static magnetic field strength B0, the Larmor frequency for imaging increases with a consequent decrease in the wavelength. For high field applications, the wavelength of the imaging frequency is larger (or comparable) than the sample dimensions, which is not the case for ultra-high field applications. In the birdcage coils, this shortening in the wavelength disturbs the sinusoidal distribution of the dominant resonance mode current, while the quadrature wavelength also approaches the length of the birdcage coil legs, which results in the formation of standing waves. The consequence of these effects is the non-homogeneous distribution of the transverse magnetic field B1. Seo et al. analyzed all three configurations (LP, HP, BP) of the birdcage coil in the presence of biological sample on various field strengths. He concluded that the birdcage coil that is implemented with higher number of legs can increase the B1 magnetic field homogeneity at ultra-high field MRI applications [71]. In a study, Nabetani et al. revealed that the utilization of the shorter legs can enhance the performance of the birdcage coil for ultra-high field MRI applications [117]. Heo et al. used the idea of shorter legs and devised a method for using an array of the birdcage coils in combination in order to improve the signal intensity and B1 magnetic field homogeneity at 7T [107].

The stray capacitance and the radiated electric field due to longer leg conductors are two such factors that become prominent at higher static field and can no longer be neglected. The stray capacitance between different conductive parts of the birdcage coil increases with the static field strength. The lumped capacitors that are inserted into the birdcage coil must be higher than the stray capacitance to avoid the shift in the resonance frequency. The radiated electric field due to longer leg conductors raises the SAR of the biological tissues, consequently inducing tissue heating. These two problems can be addressed by reducing the dimensions of the birdcage coil’s leg which resultantly reduce the coil’s inductance. The lumped capacitors in the birdcage coil are also used to serve as the electric field sinks. Breaking the leg conductors into small segments and deploying multiple series connected lumped capacitors instead of a single capacitor can be utilized to address this issue more effectively [27,118]. However, the non-linear behavior of the lumped elements at higher microwave frequencies is also an established fact. The obvious solution of the issue is to build the birdcage coils without lumped capacitors by using the distributed capacitance techniques described earlier [93,94,95].

## 5. Conclusions

The birdcage resonator is considered to be the most successful RF coil for whole volume MRI in clinical and research applications. Its excellent B1 magnetic field homogeneity and high SNR, regardless of its size, makes it a preferable choice for imaging in any field strength MRI system. A detailed literature review of the principle, functionality, design, analysis, implementation, and special types of the birdcage coil has been presented in this article. The main objective of this work was to elaborate the overall progress in all areas that are related to the birdcage coil engineering. The review of literature that is presented in this article can be summarized in three main points. First, the 3D EM simulation softwares has become an integral part in the analysis and design procedures of the birdcage RF coil. Most of the research activities that are related to the birdcage coil that has been reported in the previous decade are either completely based upon, or partially involves, the numerical electromagnetic simulations. The FEM and FDTD based 3D EM simulation software, which are commercially available, have become the preferred choice, owing to their robustness and efficiency. Second, even in the presence of advanced numerical EM simulation software, the development of analytical techniques for the simplification of the design and analysis process of the birdcage coil has become a subject of interest. The most desired and prominent feature of the analytical solutions is the establishment of the relationship between the design and analysis parameters. The maintenance of the separation between the coil leg conductors and the geometry of the conductors are considered to be critical factors in the appropriate performance of the birdcage coil. The third and most important point is the progress in the prototype implementation of the birdcage coil. The performance characteristics of the cylindrical and rectangular cross-sectional conductors at various field strengths have been the subject of debate. Moreover, it has been observed that the cylindrical conductors for low-field and rectangular conductors for high-field applications have been common choices for the prototype implementation of the novel birdcage coils. However, with such choices of leg conductors, the maintenance of the appropriate separation has always been a crucial issue. Fortunately, this issue has been effectively addressed by the FPCB-etched conductor technology. This technology is the key factor behind the development of the special-type birdcage coils. Most of the conventional, modified, and multi-resonant birdcage coils that have been reported in the previous decade were implemented by using the FPCB-etched conductor pattern.

## Figures and Tables

**Table 1:** Analysis and design process of the birdcage coil using commercially available full-wave three-dimensional electromagnetic (3D EM) simulation software.

Analysis Method	Author	Year	Simulation Software	MRI System	Research Description
FEM	Dardzinski et al. [52]	1998	Maxwell	9.4T	LP birdcage with mechanically adjustable shield for inductive frequency tuning
	Solis et al. [53]	2008	FEMLAB	4T	BP birdcage coil with lesser number of legs with larger width for the knee MRI
	Neufeld et al. [54]	2009	HFSS	8.4T	LP birdcage with higher SNR in the presence of a dielectric material
	Ahmad et al. [55]	2011	HFSS	1.5T, 3T	FPCB-based sub-leg type birdcage for double resonance
	Ahmad, et al. [56]	2015	HFSS	3T	BP birdcage coil which has an integrated detuning circuit on flexible substrate
	Kozlov et al. [57]	2016	HFSS	1.5T	Effects of tuning and matching conditions on the B1 field homogeneity of the birdcage coil
	Xu et al. [58]	2017	HFSS	9.4T	Analysis of the birdcage RF coils of various cross-sectional geometry leg conductors
	Ahmad et al. [59]	2017	HFSS	1.5T	FPCB-based BP birdcage coil for small animal
	Kozlov et al. [60]	2017	HFSS	1.5T	Effects of the EM fields of the quadrature birdcage coil on the ASTM phantom
	Shan et al. [61]	2020	HFSS	3T	Double end-ring dual tune LP-LP birdcage coil for the ^31^P/^1^H MRI
	Fantasia et al. [62]	2020	HFSS	2.35T	Double end-ring dual-tuned HP-LP birdcage coil for ^23^Na/^1^H MRI
	Gurler et al. [63]	2015	COMSOL	3T, 7T	Design and analysis of LP and HP birdcage coils.
	Fong et al. [64]	2017	COMSOL		Finding the most appropriate simulation setup for the birdcage coil analysis
	Garcia et al. [65]	2019	COMSOL	7T	Shielded HP birdcage coil for small animal MRI
FDTD	Wang et al. [31]	2006	XFDTD	3T, 7T	LP birdcage for parallel imaging by using higher order modes.
	Soe et al. [66]	2007	XFDTD	3T	Comparison of the B1 magnetic field homogeneity between the HP birdcage coil and phased array coil
	Wang et al. [67]	2012	XFDTD	7T	Multinucleus imaging with the birdcage coil
	Giovannetti et al. [68]	2015	XFDTD	3T	Dual-tune LP birdcage coil with alternate legs tuned for the ^23^Na/^1^H MRI of human calf
	Seo et al. [69]	2015	XFDTD	4.7T, 7T, 11.7T	Analysis of the birdcage coil for number of legs vs. the resulting B1 magnetic field
	Lucano et al. [70]	2016	XFDTD	1.5T	Analysis of five similar birdcage coils with different configurations and polarizations
FDTD	Seo et al. [71]	2016	XFDTD	1.5T, 3T, 4.7T, 7T, 9.4T, 11.7T	Analysis of the birdcage coils under shielding and biological subject loading conditions
	Martin et al. [72]	2016	XFDTD	7T	Analysis of the SAR as a function of legs of the birdcage coil
	Tomas et al. [73]	2013	CST	7T	Design and analysis of LP the birdcage coil
	Basari et al. [74]	2015	CST	3T	HP birdcage coil without lumped capacitors
	Byun et al. [75]	2017	CST	7T	Design and analysis of the birdcage coil inductively coupled with 16-ch array coil
	Valikovic et al. [76]	2017	CST	7T	Whole-body transmitter birdcage coil for ^31^P MRI
	Sonawane et al. [77]	2018	CST	1.5T	HP birdcage for knee MRI
	Manko et al. [78]	2019	CST	7T	LP birdcage coil for small animal MRI
	Heo et al. [79]	2020	Sim4Life	7T	Artificial circular polarization using coaxial overlapped birdcage coils
	Thiyagarajan et al. [80]	2014	EMPro	1.5T	Microstrip type HP birdcage coil for head MRI
	Seo et al. [81]	2015	XFDTD	7T	Double layer birdcage coil implemented by wounding a layer of crisscrossed loop
FIT	Gagliardi et al. [82]	2018	CST	7T	Degenerated FP birdcage coil for SAR prediction in human knee
FEM/FDTD	Reza et al. [83]	2007	HFSS XFDTD	7T	Design and analysis of the birdcage coil using HFSS for S11 and XFDTD for SAR
MoM/FEM	Lopez Rios et al. [84]	2018	FEKO	7T	BP birdcage coil for the whole-body NMR imaging of medium size animal

**Table 2:** Modified designs of the birdcage radio frequency (RF) coil.

Author	Year	MRI System	Imaging Element	Design Description
Hayes et al. [96]	1986	1.5T	^23^Na	Birdcage coil with full conductive endcap as one end-ring and a shorter endcap with the other end-ring
Harpen et al. [97]	1991	0.5T	^1^H	Spherical birdcage resonator for homogeneous B1 magnetic field in spherical volume
Li et al. [98]	1997	3.0T	^1^H	Elliptical birdcage for higher SNR
Alosp et al. [99]	1998	4.0T	^1^H	Spiral-leg birdcage coil for the improvement of B1 magnetic field homogeneity in the transverse plane for high field MRI
Pak et al. [100]	2000	3.0T	^1^H	Hybrid-leg (straight + spiral) birdcage coil to improve the B1 magnetic field homogeneity
Gulsen et al. [101]	2002	3.0T	^1^H	Birdcage coil with full conductive end caps on both sides for higher SNR and stronger B1 magnetic field
Ryang et al. [102]	2003	1.5T	^1^H	Birdcage coil with strain case-shaped legs for higher SNR and B1 magnetic field
De Zanche et al. [103]	2008	1.5T	^3^He	Non-cylindrical birdcage for easy attachment and detachment to the patient table
Kim et al. [104]	2013	1.5T	^1^H	Birdcage coil with modified legs to address the issues of a spiral birdcage
Kim, et al. [105]	2016	7T	^1^H	Asymmetric HP birdcage for superior B1 at the center of the small mouse
Xu et al. [106]	2017	9.4T	^1^H	Replacement of strip with multiple parallel wires for each leg for higher SNR and B1 magnetic field
Heo et al. [107]	2019	7T	^1^H	Two coaxial birdcage coil array configurations for the B1 magnetic field homogeneity

**Table 3:** Various techniques for the dual-resonant operation of the birdcage coils.

Author	Year	MRI System	Imaging Element	Dual Resonance Technique
Joseph et al. [39]	1989	1.9T	^19^F/^1^H	Unequal capacitances in the end-ring with specific increasing and decreasing order
Rath et al. [110]	1990	4.7T	^31^P/^1^H	Insertion of trap circuits into the end-rings and/or legs
Fitzsimmons et al. [111]	1993	2.0T	^31^P/^1^H	Two birdcage coils set up with inner low pass and outer high pass
Murphy-Boesch et al. [112]	1994	1.5T	^31^P/^1^H	Birdcage coil with four end-rings in the LP/HP and LP/LP configurations
Matson et al. [113]	1999	1.5T	^31^P/^1^H	Birdcage coil with different impedances in alternate legs
Sheikh et al. [114]	2015	4.7T	^31^P/^1^H	Utilization of dominant leg and end-ring resonances
Ahn et al. [115]	2018	4.7T	^31^P/^1^H ^23^Na/^31^P	Splitting of each leg into two different impedance sub-legs

## References

[1] Rabi I.I. (1936). On the process of space quantization. Phys. Rev..

[2] Rabi I.I. (1937). Space quantization in a gyrating magnetic field. Phys. Rev..

[3] Alvarez L.W., Bloch F. (1940). A quantitative determination of the neutron moment in absolute nuclear magnetons. Phys. Rev..

[4] Bloch F. (1946). Nuclear induction. Phys. Rev..

[5] Hoult D.I., Richards R.E. (1976). The signal-to-noise ratio of the nuclear magnetic resonance experiment. J. Magn. Reson..

[6] Damadian R. (1971). Tumor detection by nuclear magnetic resonance. Science.

[7] Lauterbur P.C. (1973). Image formation by induced local interactions: Examples employing nuclear magnetic imaging. Nature.

[8] Mansfield P., Grannell P.K. (1973). NMR ‘diffraction’ in solids?. J. Phys. C: Solid State Phys..

[9] Pykett I.J., Newhouse J.H., Buonanno F.S., Brady T.S., Goldman M.R., Kistler J.P., Pohost G.M. (1982). Principles of nuclear magnetic resonance imaging. Radiology.

[10] Pykett I.L. (1983). Instrumentation for nuclear magnetic resonance imaging. Semin. Nucl. Med..

[11] Pykett I.L., Buonanno F.S., Brady T.J. (1983). Techniques and approaches to proton NMR imaging of the head. Comput. Radiol..

[12] Aygun E., Zengin M. (1986). Nuclear Magnetic Resonance Imaging in Biomedicine. Commun. Fac. Sci. Univ. Ank. A2.

[13] Gruber B., Froeling M., Leiner T., Klomp D.W.J. (2018). RF coils: A practical guide for nonphysicists. J. Magn. Reason. Imaging.

[14] Vaughan T., Griffiths J.R. (2012). RF Coils for MRI.

[15] Mispelter J., Lupu M., Briguet A. (2015). NMR Probeheads for Biophysical and Biomedical Experiments: Theoretical Principles and Practical Guidelines.

[16] Hasse A., Odoj F., Kienline M.V., Warnking J., Fidler F., Weisser A., Nittka M., Rommel E., Lanz T., Kalusche B. (2000). NMR probeheads for in vivo applications. Concepts. Magn. Reason..

[17] Hanssum H. (1984). The magnetic field of saddle-shaped coils. I. Symmetry of the magnetic field around the coil centre. J. Phys. D Appl. Phys..

[18] Hayes C.E., Edelstein W.A., Schenck J.F., Mueller O.M., Eash M. (1985). An efficient, highly homogeneous radiofrequency coil for whole body NMR imaging at 1.5T. J. Magn. Reason..

[19] Vaughan J.T., Hetherington H.P., Harrison J.G., Otu J.O., Pan J.W., Pohost G.M. (1994). High frequency volume coils for clinical NMR imaging and spectroscopy. Magn. Reson. Med..

[20] Hayes C.E., Hattes N., Roemer P.B. (1991). Volume imaging with MR phased arrays. Magn. Reson. Med..

[21] Ackerman J.J.H., Grove T.H., Wong G.G., Gadian D.G., Radda G.K. (1980). Mapping of metabolites in whole animals by ^31^P NMR using surface coils. Nature.

[22] Fujita H., Zheng T., Yang X., Finnerty M.J., Handa S. (2013). RF surface receiver array coils: The art of an LC circuit. J. Magn. Reson. Imaging.

[23] Chen C.N., Hoult D.I., Sank V.J. (1983). Quadrature detection coils—A further √2 improvement in sensitivity. J. Magn. Reason..

[24] Mispelter J., Lupu M. (2008). Homogeneous resonators for magnetic resonance: A review. C. R. Chim..

[25] Omar A., Caverly R., Doherty W.E., Watkins R., Gopinath A., Vaughan J.T. (2011). A microwave engineer’s view of MRI. IEEE Microw. Mag..

[26] Frass-Kriegl R., Navarro de Lara L.I., Pichler M., Sieg J., Moser E., Windischberger C., Laistler E. (2018). Flexible 23-channel coil array for high-resolution magnetic resonance imaging at 3 Tesla. PLoS ONE.

[27] Hayes C.E. (2009). The development of the birdcage resonator: A historical perspective. NMR Biomed..

[28] Edelstein W.A., Schenck J.F., Mueller O.M., Lake B., Hayes C.E. (1987). Radio Frequency Coil for NMR. US Patent.

[29] Wong E.C., Luh W.-M. A multimode, single frequency birdcage coil for high sensitivity multichannel whole volume NMR imaging. Proceedings of the International Society for Magnetic Resonance in Medicine.

[30] Lin F.H., Kwong K.K., Huang I.J., Belliveau J.W., Wald L.L. (2003). Degenerate mode birdcage volume coil for sensitivity-encoded imaging. Magn. Reson. Med..

[31] Wang C., Qu P., Shen G.X. (2006). Potential advantage of higher-order modes of birdcage coil for parallel imaging. J. Magn. Reason..

[32] Alagappan V., Nistler J., Adalsteinsson E., Setsompop K., Fontius U., Zelinski A., Vester M., Wiggins G.C., Hebrank F., Renz W. (2007). Degenerate mode band-pass birdcage coil for accelerated parallel excitation. Magn. Reson. Med..

[33] Webb A.G., Smith N.B., Aussenhofer S., Kan H.E. (2011). Use of tailored higher modes of a birdcage to design a simple double-tuned proton/phosphorus coil for human calf muscle studies at 7 T. Concepts Magn. Reson. B..

[34] Jin J., Neuman M.R. (1999). Analysis and design of RF coils. Electromagnetic Analysis and Design in Magnetic Resonance Imaging.

[35] Leifer M.C. (1997). Resonant modes of the birdcage coil. J. Magn. Reason..

[36] Kim Y.C., Kim H.D., Yun B.J., Ahmad S.F. (2020). A simple analytical solution for the designing of the birdcage RF coil used in NMR imaging applications. Appl. Sci..

[37] Omar A. (2019). Design consideration for radiofrequency whole-body and head coils. IEEE J. Electromagnons. RF Microw. Med. Biol..

[38] Tropp J. (1989). The theory of bird-cage resonator. J. Magn. Reson..

[39] Joseph P.M., Lu D. (1989). A technique for double resonant operation of the birdcage imaging coils. IEEE Trans. Med. Imaging.

[40] Giovannetti G., Landini L., Santarelli M.F., Positano V. (2002). A fast and accurate simulator for the design of birdcage coils in MRI. Magn. Reason. Mat. Phys. Biol. Med..

[41] Novikov A. (2011). Advanced theory of driven birdcage resonator with losses for biomedical magnetic resonance imaging and spectroscopy. Magn. Reson. Imaging.

[42] Benyahia N., Latreche M.E. Hybrid method to compute the magnetic field in bird cage coil for a magnetic resonance imaging system. Proceedings of the Progress in Electromagnetic Research Symposium.

[43] Boissoles P., Caloz G. Accurate Calculation of Mutual Inductance and Magnetic Fields in a Birdcage Coil. https://hal.archives-ouvertes.fr/hal-00018964/document.

[44] Watkins J.C., Fukushima E. (1988). High-pass bird-cage coil for nuclear-magnetic resonance. Rev. Sci. Instrum..

[45] Pascone R., Garcia B.J., Fitzgerald T.M., Vullo T., Zipagan R., Cahill P.T. (1991). Generalized electrical analysis of low-pass and high-pass birdcage resonators. Magn. Reason. Med..

[46] Kim H.D. (2017). Analysis of the bird-cage receiver coil for MRI system employing a equivalent circuit model based on transmission matrix. J. Korea Multimed. Soc..

[47] Jin J.M., Chen J., Chew W.C., Gan H., Magin R.L., Dimbylow P.J. (1996). Computation of electromagnetic fields for high-frequency magnetic resonance imaging applications. Phys. Med. Biol..

[48] Jin J., Chen J. (1997). On the SAR and field inhomogeneity of birdcage coils loaded with the human head. Magn. Reson. Med..

[49] Chen J., Feng Z., Jin J.M. (1998). Numerical simulation of SAR and B_1_-field inhomogeneity of shielded RF coils loaded with the human head. IEEE Trans. Biomed. Eng..

[50] Jiao D., Jin J.M. (1999). Fast frequency-sweep analysis of RF coils for MRI. IEEE Trans. Biomed. Eng..

[51] Ibrahim T.S., Lee R., Baertlein B.A., Yu Y., Robitaille P.M. (2000). Computational analysis of the high pass birdcage resonator: Finite difference time domain simulations for high-field MRI. Magn. Reason. Imaging.

[52] Dardzinski B.J., Li S., Collins C.M., Williams G.D., Smith M.B. (1998). A birdcage coil tuned by RF shielding for application at 9.4 T. J. Magn. Reson..

[53] Solis S.E., Cuellar G., Wang R.R., Tomasi D., Rodriguez A.O. (2008). Transceiver 4-leg birdcage for high field MRI: Knee imaging. Rev. Mex. Fis..

[54] Neufeld A., Landsberg N., Boag A. (2009). Dielectric inserts for sensitivity and RF magnetic field enhancement in NMR volume coils. J. Magn. Reson..

[55] Ahmad S.F., Son H.W., Choi I.C., Kim Y.C., Chang Y.M., Kim H.D. Dual resonant RF coil for 1. 5T and 3T MRI systems employing FPCB etched sub-legs. In Proceedings of Asia Pacific Microwave Conference.

[56] Ahmad S.F., Kim Y.C., Choi I.C., Kim H.D. Birdcage type NMR receiver coil sensor with integrated detuning circuit for 3T MRI system. Proceedings of the IEEE Sensors2015.

[57] Kozlov M., Lucano E., Angelone L.M. Effects of tuning conditions on near field of MRI transmit birdcage coil at 64 MHz. Proceedings of the 38th Annual International Conference of the IEEE Engineering in Medicine and Biology Society.

[58] Xu Y., Wen Q. (2017). Comparison of 12 quadrature birdcage coils with different leg shapes at 9.4 T. Appl. Magn. Reason..

[59] Ahmad S.F., Kim H.D. (2017). FPCB-based birdcage-type receiving coil sensor for small animal ^1^H 1.5T magnetic resonance imaging system. J. Sens. Sci. Technol..

[60] Kozlov M., Angelone L.M., Kainz W. The electromagnetic fields of a 64 MHz quadrature driven birdcage coil in ASTM phantom. Proceedings of the 47th European Microwave Conference.

[61] Shan K., Duan Y. (2020). Rapid four-ring birdcage coil analysis: Design optimization for high efficiency, low interference, and improved body loading tolerance. Magn. Reason. Imaging.

[62] Fantasia M., Galante A., Maggiorelli F., Retico A., Fontana N., Monorchio A., Alecci M. (2020). Numerical and workbench design of 2.35 T double-tuned (^1^H/^23^Na) nested RF birdcage coils suitable for animal size MRI. IEEE Trans. Med. Imaging.

[63] Gurler N., Ider Y.Z. (2015). Numerical methods and software tools for simulation, design, and resonant mode analysis of radio frequency birdcage coils used in MRI. Concepts Magn. Reson..

[64] Fong J.T., Heckert N.A., Filliben J.J., Marcal P.V., Rainsberger R., Stupic K.F., Russek S.E. MRI birdcage RF coil resonance with uncertainty and relative error convergence rates. Proceedings of the International COMSOL Users’ Conference.

[65] Garcia M.M., Oliveira T.R., Papoti D., Chaim K.T., Otaduy M.C.G., Erni D., Zylka W. (2019). Experimental and numerical investigations of a small animal coil for ultra-high field magnetic resonance imaging (7T). Curr. Dir. Biomed. Eng..

[66] Seo J.H., Heo H.Y., Han B.H., Lee S.Y. Comparison of birdcage and phase array coil using FDTD for the B_1_ homogeneity in high field MRI. Proceedings of the 29th Annual International Conference of the IEEE Engineering in Medicine and Biology Society.

[67] Wang C., Li Y., Wu B., Xu D., Nelson S.J., Vigneron D.B., Zhang X. (2012). A practical multinuclear transceiver volume coil for in vivo MRI/MRS at 7 T. Magn. Reason. Imaging.

[68] Giovannetti G., Valvano G., Virgili G., Giannoni M., Flori A., Frijia F., De Marchi D., Hartwig V., Landini L., Aquaro G.D. (2015). Design and simulation of a dual-tuned ^1^H/^23^Na birdcage coil for MRS studies in human calf. Appl. Magn. Reason..

[69] Seo J., Han S., Kim K. (2015). Investigation of the B1 field distribution and RF power deposition in a birdcage coil as functions of the number of coil legs at 4.7 T, 7.0 T, and 11.7 T. J. Korean Phys. Soc..

[70] Lucano E., Liberti M., Mendoza G.G., Lloyd T., Iacono M.I., Apollonio F., Wedan S., Kainz W., Angelone L.M. (2016). Assessing the electromagnetic fields generated by a radiofrequency MRI body coil at 64 MHz: Defeaturing versus accuracy. IEEE Trans. Biomed. Eng..

[71] Seo J.H., Ryu Y., Han S.D., Song S., Kim H.K., Kim K.N. (2016). Influence of biological subject, shielding cage, and resonance frequency on radio wave propagation in a birdcage coil. Electron. Lett..

[72] Martin R., Vazquez J.F., Marrufo O., Solis S.E., Osorio A., Rodriguez A.O. (2016). SAR of a birdcage coil with variable number of rungs at 300 MHz. Measurement.

[73] Tomas B.P., Li H., Anjum M.R. (2013). Design and simulation of a birdcage coil using CST studio site for 7T. IOP Conf. Ser. Mater. Sci. Eng..

[74] Basari, Priatna A., Rahardjo E., Zulkifli F.Y. Numerical design of RF birdcage coil without lumped elements for MRI 3T system. Proceedings of the 14th International Conference on Quality in Research.

[75] Byun J., Seo J., Kang T., Ryu Y., Kim K. (2017). Birdcage coil with inductively coupled RF coil array for improving |B_1_|-field sensitivity in 7-T MRI. J. Magn..

[76] Valikovic L., Dragonu I., Almujayyaz S., Batzakins A., Young L.A.J., Purvis L.A.B., Clarke W.T., Wichmann T., Lanz T., Neubauer S. (2017). Using a whole-body ^31^P birdcage transmit coil and 16 element receiver array for human cardiac metabolic imaging at 7T. PLoS ONE.

[77] Sonawane S., Bhuiya T.K., Harsh R. Radiofrequency knee coil for MR application. Proceedings of the International Conference on Advances in Communication and Computing Technology.

[78] Manko M. (2019). Electromagnetic simulation of low-pass birdcage coil. MATEC Web. Conf..

[79] Heo P., Kim H., Kim S., Kim D., Kim K. (2020). Computational analysis of overlapping birdcage coil for artificial circular-polarized mode in magnetic resonance imaging. J. Korean Phys. Soc..

[80] Thiyagarajan K., Kesavamurthy T., Bharathkumar G. (2014). Design and analysis of microstrip-based RF birdcage coil for 1.5 T magnetic resonance imaging. Appl. Magn. Reason..

[81] Seo J., Han S., Kim K. (2015). Design of crisscrossed double-layer birdcage coil for improving B_1_^+^ field homogeneity for small-animal magnetic resonance imaging at 300 MHz. J. Magn..

[82] Gagliardi V., Retico A., Biagi L., Aringhieri G., Zampa V., Symma M.R., Tiberi G., Tosetti M. Subject-specific knee SAR prediction using a degenerate birdcage at 7T. Proceedings of the IEEE International Symposium on Medical Measurements and Applications.

[83] Reza S., Vijayakumar S., Limkeman M., Huang F., Saylor C. (2007). SAR simulation and the effect of mode coupling in a birdcage resonator. Concepts Magn. Reson..

[84] Lopez Rios N., Pouliot P., Papoutsis K., Foias A., Stikov N., Lesage F., Dehaes M., Cohen-Adad J. (2018). Design and construction of an optimized transmit/receive hybrid birdcage resonator to improve full body images of medium-sized animals in 7T scanner. PLoS ONE.

[85] Li B.K., Liu F., Weber E., Crozier S. (2009). Hybrid numerical techniques for the modelling of radiofrequency coils in MRI. NMR Biomed..

[86] Rao M.R., Stewart N.J., Griffiths P.D., Norquay G., Wild J.M. (2018). Imaging human brain perfusion with inhaled hyperpolarized ^129^Xe MR imaging. Radiology.

[87] Giovannetti G., Francesconi R., Landini L., Santarelli M.F., Positano V., Viti V., Benassi A. (2004). Conductor geometry and capacitor quality for performance optimization of low-frequency birdcage coils. Concepts Magn. Reason. B.

[88] Ahmad S.F., Kim Y.C., Choi I.C., Choi S.W., Kim T.G., Ahn C.M., Kim H.D. Fast and efficient birdcage coil design process for high field MRI system by combining equivalent circuit model and 3D electromagnetic simulation. Proceedings of the Asia Modelling Symposium.

[89] Tropp J. (1997). Mutual inductance in the bird-cage resonator. J. Magn. Reson..

[90] Chin C.L., Collins C.M., Li S., Dardzinski B.J., Smith M.B. (2002). Birdcage builder: Design of specified geometry birdcage coils with desired current pattern and resonant frequency. Concepts Magn. Reason..

[91] Pascone R., Vullo T., Farrely J., Cahill P.T. (1992). Explicit treatment of mutual inductance in eight-column birdcage resonators. Magn. Reson. Med..

[92] Son H.W., Ahmad S.F., Choi J.Y., Kim H.D., Cho Y. Effect of distributed capacitance on the performance of birdcage type RF coil for ^1^H MRI. Proceedings of the International Symposium on Antennas and Propagation.

[93] Su S., Saunders J.K. (1996). A new miniaturizable birdcage resonator design with improved electric field characteristics. J. Magn. Reson..

[94] Suga R., Saito K., Takahashi M., Ito K. Magnetic field distribution of birdcage coil for 4 T MRI system with no lumped circuit elements. Proceedings of the 4th International Symposium on Applied Sciences in Biomedical and Communication Technologies.

[95] Stara R., Tiberi G., Gabrieli M., Buonincontri G., Fontana N., Monorchio A., Costagli M., Symms M.R., Retico A., Tosetti M. (2015). Quadrature birdcage coil with distributed capacitors for 7.0 T magnetic resonance data acquisition of small animals. Concepts Magn. Reson..

[96] Hayes C.E. An endcap birdcage resonator for head imaging. Proceedings of the International Society for Magnetic Resonance in Medicine.

[97] Harpen M.D. (1991). The spherical birdcage resonator. J. Magn. Reson..

[98] Li S., Collins C.M., Dardzinski B.J., Chin C.L., Smith M.B. (1997). A method to create an optimum current distribution and homogeneous B_1_ field for elliptical birdcage coils. Magn. Reason. Med..

[99] Alsop D.C., Connick T.J., Mizsei G. (1998). A spiral volume coil for improved RF field homogeneity at high static magnetic field strength. Magn. Reson. Med..

[100] Pak J.S., Kim J.H., Lee J.O., Park B.S., Jung S.P., Jung K.J., Kim J.H. A new 3.0 T hybrid-spiral birdcage (HSB) coil for improved homogeneity along Z-axis. Proceedings of the International Society for Magnetic Resonance in Medicine.

[101] Gulsen G., Muftuler L.T., Nalcioglu O. (2002). A double end-cap birdcage RF coil for small animal whole body imaging. J. Magn. Reson..

[102] Ryang K.S., Shin Y.J. (2003). Fabrication of a straincase coil with improved SNR and image uniformity by structural changes of a conventional birdcage coil at 1.5T MRI. J. Korea Magn. Reson..

[103] De Zanche N., Chhina N., Teh K., Randell C., Pruessmann K.P., Wild J.M. (2008). Asymmetric quadrature split birdcage coil for hyperpolarized ^3^He lung MRI at 1.5T. Magn. Reason. Med..

[104] Kim J.M., Ahmad S.F., Choi I.C., Kim Y.C., Kim H.D. Saw-tooth shaped legs birdcage RF coil for small animal NMR imaging at 1.5T MRI systems. In Proceedings of Asia Pacific Microwave Conference.

[105] Kim K.N., Han S.D., Seo J.H., Heo P., Yoo D.K., Im G.H., Lee J.H. (2017). An asymmetric birdcage coil for small-animal MR imaging at 7T. Magn. Reason. Med. Sci..

[106] Xu Y., Wen Q., Yang H., Zhong K. (2018). Multiple parallel round leg design for quadrature birdcage coil in ultrahigh-field MRI. Appl. Magn. Reason..

[107] Heo P., Kim H.J., Han S.D., Kim D.H., Im G.H., Kim S.B., Kim K.N. (2020). A study on multiple array method of birdcage coils to improve the signal intensity and homogeneity in small-animal whole-body magnetic resonance imaging at 7T. Int. J. Imaging Syst. Technol..

[108] Pimmel P., Briguet A. (1992). A hybrid bird cage resonator for sodium observations at 4.7T. Magn. Reson. Med..

[109] Loring J., Van der Kemp W.J.M., Almujayyaz S., Van Oorschot J.W.M., Luijten P.R., Klomp D.W. (2016). Whole-body radiofrequency coil for ^31^P MRSI at 7T. NMR Biomed..

[110] Rath A.A. (1990). Design and performance of a double-tuned bird-cage RF coils. J. Magn. Reson..

[111] Fitzsimmons J.R., Beck B.L., Brooker H.R. (1993). Double resonant quadrature birdcage. Magn. Reason. Med..

[112] Murphy-Boesch J., Srinivasan R., Carvajal L., Brown T.R. (1994). Two configurations of the four-ring birdcage coil for ^1^H imaging and ^1^H-decoupled ^31^P spectroscopy of the human head. J. Magn. Reson. B.

[113] Matson G.B., Vermathen P., Hill T.C. (1999). A practical double-tuned ^1^H/^31^P quadrature birdcage headcoil optimized for ^31^P operation. Magn. Reson. Med..

[114] Sheikh F.A., Kim Y.C., Choi I.C., Kim H.D. (2015). Double tuned RF receiver coil for detecting both ^1^H and ^31^P elements in 4.7 T MRI system. Electron. Lett..

[115] Ahn C.M., Sheikh F.A., Kim Y.C., Kim H.D. Double band-pass birdcage RF coil for dual elements NMR imaging at 4.7T MRI system. Proceedings of the IEEE International RF and Microwave Conference.

[116] Kathiravan S., Kanakaraj J. (2013). A review on potential issues and challenges in MR imaging. Sci. World J..

[117] Nabetani A., McKinnon G., Nakada T. Performance comparison with 15cm long and 23cm long birdcage coil on 7T. Proceedings of the International Society for Magnetic Resonance in Medicine.

[118] Hayes C.E., Mathis C.M. A distributed capacitor endcap for head coils at 1.5T and 3.0T. Proceedings of the International Society for Magnetic Resonance in Medicine.

